# Adipokines, Metabolic Syndrome and Rheumatic Diseases

**DOI:** 10.1155/2014/343746

**Published:** 2014-02-26

**Authors:** Vanessa Abella, Morena Scotece, Javier Conde, Verónica López, Verónica Lazzaro, Jesús Pino, Juan J. Gómez-Reino, Oreste Gualillo

**Affiliations:** ^1^SERGAS, Research Laboratory 9, NEIRID Lab (Neuroendocrine Interactions in Rheumatology and Inflammatory Diseases), Institute of Medical Research (IDIS), Santiago University Clinical Hospital, 15706 Santiago de Compostela, Spain; ^2^Department of Molecular and Cellular Biology, University of Coruña (UDC), 15071 A Coruña, Spain; ^3^University Magna Graecia of Catanzaro, 88100 Catanzaro, Italy; ^4^SERGAS, Division of Orthopaedics Surgery and Traumatology, Santiago University Clinical Hospital, 15706 Santiago de Compostela, Spain

## Abstract

The metabolic syndrome (MetS) is a cluster of cardiometabolic disorders that result from the increasing prevalence of obesity. The major components of MetS include insulin resistance, central obesity, dyslipidemia, and hypertension. MetS identifies the central obesity with increased risk for cardiovascular diseases (CVDs) and type-2 diabetes mellitus (T2DM). Patients with rheumatic diseases, such as rheumatoid arthritis, osteoarthritis, systemic lupus erythematosus, and ankylosing spondylitis, have increased prevalence of CVDs. Moreover, CVD risk is increased when obesity is present in these patients. However, traditional cardiovascular risk factors do not completely explain the enhanced cardiovascular risk in this population. Thus, MetS and the altered secretion patterns of proinflammatory adipokines present in obesity could be the link between CVDs and rheumatic diseases. Furthermore, adipokines have been linked to the pathogenesis of MetS and its comorbidities through their effects on vascular function and inflammation. In the present paper, we review recent evidence of the role played by adipokines in the modulation of MetS in the general population, and in patients with rheumatic diseases.

## 1. Introduction

The metabolic syndrome (MetS) is a cluster of cardiometabolic disorders that result from the increasing prevalence of obesity. The major components of MetS include insulin resistance, central obesity, dyslipidemia, and hypertension [1]. It is widely accepted that the concept of MetS identifies the central obesity with increased risk for cardiovascular diseases (CVDs) and type-2 diabetes mellitus (T2DM) [2, 3]. Nevertheless, it still lacks a universally accepted definition. Various diagnostic criteria have been proposed by different organizations over the past decade, thus giving five definitions for MetS [4–8]. Recently, the International Diabetes Federation (IDF) and the American Heart Association/National Heart, Lung, and Blood Institute (AHA/NHLBI) have developed one unified definition [9]. Several rheumatic diseases (rheumatoid arthritis (RA), osteoarthritis (OA), systemic lupus erythematosus (SLE), and ankylosing spondylitis (AS)) have been associated with an increase in the prevalence of CVDs. Though traditional cardiovascular risk factors (sex, smoking, dyslipidemia, age, and hypertension) have been involved in the pathogenesis of CVDs in patients with rheumatic diseases, these features do not completely explain the enhanced cardiovascular risk in this population [10]. For instance, CVDs are responsible for almost 50% excess of mortality in patients with RA [11, 12]. Furthermore, the incidence of CVDs is increased when obesity is present in patients with rheumatic disorders. Evidence now indicates that MetS begins with excess of central adiposity [13]. Thus, MetS and obesity, in particular, pathologic dysfunction of fat mass, due to altered secretion patterns of proinflammatory adipokines, could be the link between CVDs and rheumatic diseases [14, 15]. Adipose tissue is now recognized as an endocrine organ able to secrete adipose-derived factors named adipokines. Adipokines have been linked to the pathogenesis of MetS and its comorbidities through their effects on vascular function and inflammation [14, 16, 17]. The present paper reviews recent lines of evidence of the role played by adipokines produced by adipose tissue in the modulation of MetS in the general population and in patients with rheumatic diseases.

## 2. Metabolic Syndrome and Rheumatic Diseases

**Figure 1:**
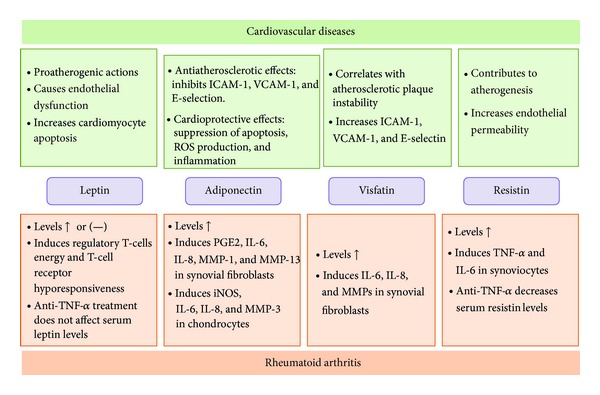
Schematic representation of the relations between adipokines, cardiovascular diseases, and rheumatoid arthritis.

Patients with chronic rheumatic diseases have an increased risk for CVDs morbidity and mortality but the pathogenetic factors involved are not yet fully understood. MetS may provide an additional link between accelerated atherosclerosis and inflammation in these diseases [2] (Figure 1).

The great majority of these studies demonstrated that the prevalence of MetS is higher in rheumatological diseases than in the control populations, suggesting that either the presence or the treatment of those diseases seems to influence the risk of developing metabolic syndrome [18]. MetS is not uncommon in patients with RA [19]. The risk of having moderate-to-severe RA was higher in patients with MetS than in those without MetS, and the disease activity correlated with the number of MetS parameters present. Thus, MetS might have inflammatory milieu leading to the occurrence of more severe RA [20]. In studies evaluating the association of MetS and RA, the prevalence of MetS is higher in RA subjects, but the values vary according to the MetS criteria used [21, 22]. A different approach was developed by Crowson et al. The authors noticed that a more clinically relevant question was whether the prevalence of MetS is increased in RA subjects without overt CVD. In fact, they observed higher prevalence of MetS in RA subjects (33%) compared with non-RA subjects (25%) [23].

The increase of fat mass is also related to the occurrence of OA and to the plethora of cardiovascular comorbidities. Regarding the influence of fat mass on OA, it is evident that biomechanical aspects are of weight in the pathogenesis of diseases. Biomechanical loading is necessary for the maintenance of cartilage homeostasis. However, abnormal loading is associated with inflammatory and metabolic imbalances, in part because it triggers the same signaling pathways as those induced by inflammatory cytokines [24]. Chondrocytes sense mechanical stress through ion channels, integrin-mediated connections to the extracellular matrix, and intracellular or membrane deformation [25]. However, OA is more common in women [26, 27] and exists in non-weight-bearing joints [28], indicating that a metabolic component is also present [29]. Of late, OA and MetS have been related to each other. The prevalence of MetS is higher in OA subjects [30, 31]. Recently, Zhuo et al. proposed a theory focused on inflammation, oxidative stress, common metabolites, and endothelial dysfunction to link metabolic OA aetiologically to MetS. They suggest that metabolic OA should be a new facet of the definition of MetS, supported by its strong associations and shared mechanisms with MetS components. However, further research is needed to define the reciprocal influence of OA on the currently accepted components of MetS [32].

A link between SLE and MetS also exists. Most of the reports showed major prevalence of MetS in lupus patients than in healthy controls. MetS is also common in young patients with recently diagnosed SLE [33]. Moreover, subjects with SLE and MetS presented higher levels of inflammatory markers than SLE without MetS [34–36]. SLE patients also have a higher risk of experiencing CVDs and metabolic disorders related to MetS may contribute to overall CVD risk [37].

The link between MetS and rheumatic diseases is also at play in AS. It has been reported that AS patients have risk of experiencing MetS and CVDs than in healthy controls (45.8% versus 10.5%) [38], even after receiving anti-tumor necrosis factor (anti-TNF) therapy [39]. Moreover, in these patients, MetS was associated with higher disease activity.

Several features of MetS, such as dyslipidemia, have also been reported, with higher prevalence in Sjögren's syndrome. It is noteworthy that metabolic alterations were associated with a differentiated pattern of clinical and immunological Sjögren's syndrome expression but not with Sjögren's syndrome-related therapies (except for the higher frequency of T2DM observed in patients treated with corticosteroids) [40].

## 3. Adipokines and Metabolic Syndrome

White adipose tissue (WAT) is described as an endocrine organ, which secretes a wide variety of factors called adipokines. Adipokines are pleiotropic molecules that contribute to the so-called low-grade inflammatory state of obese subjects creating a cluster of metabolic aberrations including autoimmune and inflammatory diseases that affect joints and bone [41, 42]. Recent studies show a potential source of adipokines at articular level: the infrapatellar fat pad (IPFP). Actually, recent evidence indicates an inflammatory phenotype of this adipose compartment in patients with OA showing that IPFP could contribute to the pathophysiological changes in the OA joint via the local production of cytokines and adipokines [43]. In the majority of obese patients, a dysfunctional adipose tissue mechanistically links obesity to other manifestations such as CVDs and MetS. This dysfunction is caused by complex disequilibrium between genetic and environmental factors, which is characterized by adipocyte hypertrophy, hypoxia, and inflammation. The direct consequence of this dysfunction is that adipokine secretion is shifted to an atherogenic, diabetogenic, and proinflammatory secretion pattern. According to the recent literature, all known adipokines are markedly dysregulated when abnormal abdominal fat accumulation is present, thereby promoting metabolic and cardiovascular disorders (Figure 1). Adipokines were recently proposed as novel biomarkers and regulators of MetS, given the association of adipokines plasma concentration and MetS [44]. Among the different adipokines, leptin and adiponectin were identified as relevant factors involved in interactions between metabolism and rheumatic disorders. Recent research suggests that leptin may be an important factor linking obesity, the metabolic syndrome, and cardiovascular disorders [45]. Additionally, several clinical observations support an association between adiponectin levels and obesity-related metabolic dysfunction [46]. Given the recent pieces of evidence about the influence of adipokines in both metabolic disorders and rheumatic diseases, here we present an update summary of the main fat mass induced adipokines as key players in MetS and the most relevant rheumatic diseases.

## 4. Leptin

Leptin is a 16 kDa nonglycosylated hormone that belongs to the class 1 cytokine superfamily. It is encoded by the obese (*ob*) gene, the murine homolog of human LEP gene [47]. Leptin exerts its biological actions through the activation of OB-Rb long-form isoform receptors encoded by the diabetes (*db*) gene. It is mainly produced by adipocytes, and its circulating levels positively correlate with WAT mass and body mass index (BMI). Leptin levels are mostly dependent on the amount of body fat, but its synthesis is also regulated by inflammatory mediators [48]. It decreases food intake and increases energy consumption by acting on specific hypothalamic nuclei, inducing anorexigenic factors and suppressing orexigenic neuropeptides [49].

### 4.1. Leptin and Metabolic Syndrome

**Figure 2:**
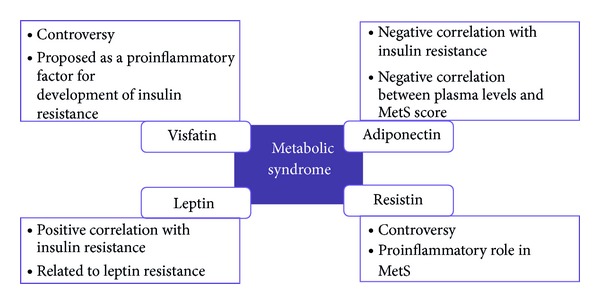
Schematic representation of adipokines actions in metabolic syndrome.

Leptin was associated with MetS. Subjects with MetS had higher leptin levels compared with individuals without MetS. Furthermore, leptin was strongly correlated with waist circumference and insulin sensitivity [50]. Quercioli et al. observed that the decrease in leptin plasma levels with decreasing body weight was not observed to be related to the improvement in coronary circulatory function [51]. This observation may emphasize that beneficial effect of leptin plasma levels on endothelium-related coronary function may operate predominantly in morbidly obese individuals with a sevenfold increase of its concentration to counterbalance the adverse effects of obesity on coronary circulatory dysfunction as they are previously described [52]. Noteworthy, leptin levels predict MetS development independent of obesity. This association is specifically related to the development of glucose intolerance and insulin resistance [53]. Recent works support this relationship. Chiu et al. have observed how leptin concentrations varied in relation to MetS score in both genders in Taiwanese adults [54]. In another study, leptin showed significant positive correlation with parameters of insulin resistance and with triglycerides and strong negative correlation with HDL-cholesterol [55]. Insulin resistance and changes in lipid parameters are typical for early signs of MetS. Kontunen et al. assessed leptin levels in patients with arthritis and MetS. Their results showed higher leptin levels in subjects with arthritis and MetS than arthritis patients without MetS [56]. This suggests that leptin is associated with MetS but not directly with arthritis, although a marked increase in plasma levels of leptin in patients with RA was noted [57]. Leptin resistance is believed to be a major factor leading to MetS. The term leptin resistance is used to describe the failure of obesity-related hyperleptinemia to reduce food intake and increase energy expenditure, at which point MetS is present [58] (Figure 2).

### 4.2. Leptin and Rheumatic Diseases

Leptin has been associated with rheumatic diseases due to its ability to modulate bone and cartilage metabolism [59, 60]. This adipokine plays main role in certain autoimmune diseases such as rheumatoid arthritis (RA). Generally, the present consensus is that leptin levels are elevated in patients with RA. However, in patients with RA undergoing anti-TNF-*α* therapy, no immediate change in serum leptin levels was observed [61] (Table 1). Several authors have also reported that the ratio between serum leptin levels and the synovial fluid (SF) is correlated with disease duration and parameters of RA activity [62]. Leptin has been supposed to play a part in RA although it is still unclear whether leptin can damage or protect joint structures in RA. In fact, this hormone, generally considered to be proinflammatory, has also been reported to be associated with reduced radiographic joint damage [63], and this effect could be related to anabolic effects of leptin [64]. The effects of leptin in RA are not only related to articular tissues. Leptin also modulates the activity of multiple immune cells [65]. The ability of leptin to induce regulatory T-cells anergy and T-cell receptor hyporesponsiveness has gained much interest since altered functioning of this cell type was described in RA [66].

Leptin has also been related to osteoarthritis (OA) and cartilage metabolism. Recently, the NEIRID group showed that leptin expression was higher in infrapatellar fat pad (IPFP) and synovial tissues obtained from OA patients compared to healthy controls [43]. Furthermore, chondrocytes from human OA cartilage produce much more leptin than those from normal cartilage [64]. In fact, the expression pattern of leptin was related to the grade of cartilage destruction [64], with the highest levels of leptin in the advanced stages of the disease [72, 73]. Leptin could perpetuate cartilage-degrading processes by inducing VCAM-1 expression, adhesion molecule responsible for leukocyte and monocyte infiltration at inflamed joints [74]. In addition, leptin induces IL-8 secretion in human primary chondrocytes contributing to the chemotactic gradient seen in inflamed joints [75]. Leptin is necessary for the development and progression of OA associated with obesity. Impaired leptin signalling associated with extreme obesity induces alterations in subchondral bone morphology but without increasing the incidence of OA [76]. These results suggest that obesity, per se, is not sufficient to induce OA.

The role of leptin in SLE is controversial. Nowadays, most of the studies suggest a role for leptin in this disease. Several authors found higher leptin levels in SLE patients compared with healthy controls, even after BMI correction [36, 77, 78]. Interestingly, in some of these studies, the hyperleptinemia was associated with CVDs and with several features of MetS [36, 78]. On the other hand, other groups have described lower or unchanged circulating leptin levels in SLE patients compared to healthy control [79, 80].

The role of leptin in ankylosing spondylitis (AS) is still unclear and the data available are almost limited. For instance, certain studies have not found any correlation between serum leptin concentrations and markers of disease activity [81, 82]. However, other authors determined an association among serum leptin levels, CRP, IL-6, and markers of disease activity [83, 84].

## 5. Adiponectin

Adiponectin is a 244-residue protein, also known as GBP28, apM1, Acrp30, or AdipoQ. It has structural homology with collagen VIII, collagen X, and complement factor C1q. Adiponectin acts via two receptors, one (AdipoR1) found predominantly in skeletal muscle and the other (AdipoR2) in liver. Transduction of the adiponectin signal by AdipoR1 and AdipoR2 involves the activation of AMPK, PPAR-*α*, PPAR-*γ*, and other signaling molecules. Adiponectin is mainly synthesized by adipose tissue in different molecular forms. Circulating adiponectin levels tend to be low in obese patients and increase with weight loss [85, 86]. It increases fatty acid oxidation and glucose uptake in the muscle and reduces the synthesis of glucose in the liver. Ablation of the adiponectin gene has no dramatic effect in knockout mice on a normal diet, but when placed on a high fat/sucrose diet, they develop severe insulin resistance and exhibit lipid accumulation in muscles [85].

### 5.1. Adiponectin and Metabolic Syndrome

Unlike most other adipokines, plasma levels of adiponectin are decreased in obesity and related pathologies, including T2DM and CVDs [87–89]. Adiponectin levels are inversely proportional to obesity and insulin resistance, increasing with weight loss and with the use of insulin-sensitizing drugs [85]. Its secretion is inhibited by proinflammatory cytokines [90], suggesting that inflammation might be an important factor contributing to hypoadiponectinemia in insulin-resistant and obese states [91]. On the other hand, physical training increases circulating adiponectin and expression of its receptors [92]. Furthermore, dyslipidemia is also associated with low circulating levels of adiponectin, even in the absence of other MetS risk factors [93]. Recently, many studies have proposed adiponectin as a MetS biomarker. Bae et al. reported a strong negative correlation between MetS score and serum levels [94] (Figure 2). In subjects with nascent MetS (without confounding T2DM and/or CVDs), adiponectin concentration was lower than in controls [95]. Kim et al., in a prospective cohort study, stated that decreasing levels of adiponectin were progressively associated with increased incidence of MetS [96]. Of late, the serum/plasma leptin: adiponectin ratio (L : A) has been reported to be associated with dyslipidemia and insulin resistance [97]. Kotani and Sakane suggested that the L : A ratio can serve as a clinically useful marker for detecting MetS characteristics in the general Japanese population [98]. From the historical cohort of the Brisighella Heart Study, Cicero et al. observed that subjects without MetS had significantly lower L : A ratio than those with MetS [99]. In other study, Kontunen et al. assessed adiponectin levels in patients with arthritis and MetS. Their results showed lower adiponectin levels in subjects with arthritis and MetS than arthritis patients without MetS [56]. Furthermore, Gonzalez-Gay et al. suggested that low circulating adiponectin levels may be implicated in the development of CVD associated with RA. They observed that in patients with RA undergoing anti-TNF-*α* therapy, high-grade inflammation was independently and negatively correlated with circulating adiponectin concentrations. However, the interaction of high-grade inflammation with low circulating adiponectin concentrations was not likely to be TNF-*α* mediated in RA [100] (Table 1).

### 5.2. Adiponectin and Rheumatic Diseases

In contrast to its previously described protective role in CVDs and obesity, there are multiple lines of evidence that adiponectin acts as a proinflammatory factor in joints and it could be involved in matrix degradation. In cultured chondrocytes, adiponectin stimulates the secretion of proinflammatory mediators (iNOS, IL-6, and IL-8) [75, 101] and increases MMP-3 expression [102]. Adiponectin levels have been found to be higher in RA patients than in healthy controls [57, 63, 67, 103, 104]. Recently, it has been reported that adiponectin and adiponectin receptor-1 expression are higher in synovial fluids and the synovial tissues of RA patients compared with controls, confirming the correlation of circulating adiponectin levels with the severity of RA [67]. Recent studies showed that adiponectin might also contribute to synovitis and joint destruction in RA by stimulating MMP-1 and MMP-13 [105]. Moreover, Frommer et al. described that the different isoforms of adiponectin can induce the expression of different genes involved in the pathogenesis of RA [106], further demonstrating that adiponectin could have detrimental effects on RA pathology.

Adiponectin is also implicated in the pathogenesis of OA. It has been reported that plasma and serum levels of adiponectin were significantly higher in OA patients than in healthy controls [107, 108] and correlate with disease severity [109]. In addition, adiponectin induces VCAM-1 expression [74] and IL-8 secretion [75] in human primary chondrocytes, contributing to, respectively, the leukocyte and monocyte infiltration and the chemotactic gradient seen in inflamed joints. However, some data analyzing the role of adiponectin in OA are controversial. Adiponectin has been shown to inhibit IL-1b-induced MMP-13 expression and to upregulate TIMP-2 production in chondrocytes [110]. Moreover, in STR/Ort mice, a spontaneous animal OA model, serum adiponectin levels were lower compared with the control group [111], suggesting a protective role for this adipokine in the development and/or progression of the disease. However, only a few clinical data support the hypothesis that adiponectin could be a protective molecule against OA. One study revealed an inverse correlation between adiponectin and disease severity [112]. Moreover, another recent study showed that serum adiponectin levels were not associated with radiographic hand OA severity [113]. These contradictory results can be explained by differences in patient characteristics and study protocols. It is also possible that the significance of adiponectin varies according to the phase and severity of the OA process.

Concerning the role of adiponectin in SLE, several studies have showed elevated levels of this adipokine in SLE patients [77, 79, 114]. Nevertheless, other authors did not find any difference in adiponectin levels between SLE patients and controls [78, 115]. However, the same authors find increased MetS prevalence among subjects with SLE and low levels of adiponectin in SLE patients with insulin resistance (IR) compared to SLE subjects without IR, confirming the results by Sada et al. [77, 78]. It also has been reported that mice with experimental lupus, that lack adiponectin, develop more severe disease than wild-type mice, suggesting the involvement of adiponectin in regulating disease activity [116].

Little is known about the role of adiponectin in other rheumatic diseases, such as AS and Sjögren's syndrome. However, it has been reported that serum adiponectin levels are not different between AS patients and healthy controls [82]. Regarding Sjögren's syndrome, it has been described that adiponectin is expressed in salivary gland epithelial cells and this expression is higher in patients with Sjögren's syndrome [117].

## 6. Visfatin

Visfatin is a protein of approximately 471 amino acids and 52 kDa, also called PBEF (pre-B cell colony enhancing factor) and NAMPT (nicotinamide phosphoribosyltransferase) [118]. It was originally discovered in liver, bone marrow, and muscle, but it is also secreted by macrophages and visceral adipose tissue [119–121]. A specific receptor for visfatin has not been identified yet. It has been reported that visfatin is increased in obesity [122]. Moreover, leucocytes from obese patients produce higher amounts of visfatin compared with lean subjects [123]. It is supposed that visfatin has insulin mimetic properties; however, the role of this adipokine in glucose metabolism is still unclear and controversial [120]. Visfatin synthesis is regulated by factors such as glucocorticoids, TNF-*α*, IL-6, and growth hormone (GH). Moreover, visfatin has been shown to induce chemotaxis and the production of IL-1*β*, TNF-*α*, and IL-6 in lymphocytes from obese patients, suggesting potential involvement in the obesity-associated inflammatory state [122].

### 6.1. Visfatin and Metabolic Syndrome

The relation of visfatin with MetS is still confusing, but some studies have addressed this issue. Several authors have shown that serum visfatin was increased in subjects diagnosed with obesity, type-2 diabetes mellitus, and MetS [124]. However, other studies have not detected this relation. De Luis et al. reported that in a sample of 826 female obese subjects, 42.4% had MetS. Their findings showed that serum visfatin was correlated with total cholesterol and C-reactive protein. However, in the multivariate analysis, only C-reactive protein remained associated with serum visfatin and serum visfatin was not associated with the accumulation of MetS factors or the diagnosis of MetS [125]. Specifically, in obese women, Olszanecka-Glinianowicz et al. did not find a relation between visfatin levels and the presence of MetS. Nevertheless, these authors suggest that the proportion of the circulating visfatin and insulin molecules, expressed as the visfatin/insulin ratio, more than visfatin alone would be a good indicator for prevention of the development of insulin resistance and MetS in the obese [126]. In another approach, Bremer and Jialal investigated the levels of visfatin in plasma and in subcutaneous fat (SAT), finding no difference between MetS and control subjects [95]. Further studies are needed to analyse this unclear topic area, because visfatin could be a proinflammatory factor favoring the development of insulin resistance, as it was proposed [127] (Figure 2).

### 6.2. Visfatin and Rheumatic Diseases

Recent findings suggest, in arthritis animal models, that visfatin might act as a relevant regulator of the inflammation and joint destruction. In fact, it has been reported that serum and SF levels of visfatin were increased in arthritis models [128–130]. Clinical data also suggest a role for visfatin in the development of RA. The NEIRID group and others demonstrated that serum visfatin concentrations were higher in RA patients compared with healthy controls [57, 63, 131, 132]. Moreover, an association between serum visfatin levels and radiographic joint damage has been described [63, 133]. By contrast, the relationship between visfatin and disease activity presents conflicting results. Although Alkady et al. reported an association between these two parameters [131], others did not find any correlation [132, 134]. Results obtained from trials treating RA patients with anti-TNF-*α* therapy also present some discrepancies. Several authors described a reduction in visfatin levels upon anti-TNF-*α* therapy [132, 135], but others did not show any variation [134] (Table 1). However, all the data presented showed that visfatin participates in RA pathology, even if the exact mechanisms by which this adipokine exerts its proinflammatory and catabolic actions are not completely understood.

At cartilage level, human OA chondrocytes produce visfatin. This adipokine increases the expression of ADAMTS4, ADAMTS5, MMP-3, and MMP-13, which are very relevant cartilage degradative enzymes [136]. Moreover, OA patients had higher synovial fluid visfatin concentrations, which are correlated with degradation biomarkers such as collagen type II and aggrecan [68]. Taken together, these data indicate that visfatin develops catabolic functions at cartilage level and it could play an important role in the pathophysiology of OA.

Studies performed in SLE and AS patients present conflicting results. Some authors determined higher visfatin levels in SLE patients than in healthy controls [114], but others did not found any variation between patients and controls [137]. Similarly, there was no association between visfatin levels and disease activity in both SLE and AS [81, 137].

## 7. Resistin

Resistin is also known as ADSF (adipocyte-secreted factor) or FIZZ3 (found in inflammatory zone 3). It is a 12.5 kDa protein that belongs to the resistin-like molecules (RELMs) family [70]. The major source of resistin in mice is white adipose tissue (WAT) [69], whereas, in humans, it is predominantly expressed in macrophages [138]. Thus, in human adipose tissue, resistin is mainly produced by nonadipocyte resident inflammatory cells [139]. Resistin receptor remains unknown, but recently TLR4 was proposed to mediate resistin inflammatory responses in human cells [140]. Serum resistin levels increase with obesity in mice, rats, and humans [141, 142]. In animal models, resistin promotes insulin resistance, while the evidence for this effect in human is less clear [70, 143], so that it was proposed as potential link between obesity and diabetes [70].

### 7.1. Resistin and Metabolic Syndrome

In humans, data on the role of this adipokine in insulin sensitivity and obesity are controversial [144]. Some authors indicated that increased serum resistin levels are associated with increased obesity, visceral fat [70], insulin resistance, and T2DM, while other groups failed to observe such correlations [145]. Recent investigations have attempted to shed light on the debate [146, 147]. As MetS by itself is associated with inflammation, there might be the possibility that resistin is rather associated with inflammation markers that would appear at different stages of MetS development and its correlation with other metabolic and anthropometric parameters like glucose, blood lipids, and BMI is just a secondary effect [148]. Whether resistin is an active player or merely a responder in metabolic dysfunction cannot be fully determined without understanding the regulation of resistin itself. Genetic determinants of resistin expression may provide additional clues about the role of resistin in human susceptibility to disease [149] (Figure 2).

### 7.2. Resistin and Rheumatic Diseases

Until now, a significant difference was not found in serum resistin levels between RA patients and healthy controls [57, 150]. However, a role for resistin has been proposed and its involvement in RA pathology was explored [151]. Recombinant resistin increases the production of several proinflammatory cytokines, and the intra-articular injection of this adipokine in the knee joints of mice causes arthritis [151]. In humans, there is also evidence for a role of resistin in RA pathology. Anti-TNF drugs reduced serum resistin levels [135, 152] (Table 1), but no association has been found between resistin concentrations and radiographic progression [133]. By contrast, there are several studies that report an association between resistin and markers of inflammation such as C-reactive protein (CRP), erythrocyte sedimentation rate (ESR), IL- 6, IL-1RA, or leukocyte count in RA patients [56, 150, 153]. Furthermore, resistin has been correlated with disease activity and joint destruction [153], and synovial fluid samples from RA patients showed higher levels of this adipokine than those from OA patients [151, 153], suggesting that resistin is produced in the inflamed joint.

## 8. Other Adipokines

### 8.1. Lipocalin-2

Lipocalin-2 (LCN2), also known as siderocalin, 24p3, uterocalin, and neutrophil gelatinase-associated lipocalin (NGAL), is a 25 kDa glycoprotein isolated from neutrophil granules, although WAT is thought to be the main source [154]. This adipokine is believed to bind small lipophilic substances, such as steroids and LPS [155], and has been reported to have roles in the induction of apoptosis in hematopoietic cells [156], transport of fatty acids [71], modulation of inflammation [157], and metabolic homeostasis [158]. LCN2 expression is altered in several pathologic conditions, such as adipose tissue hypoxia and obesity [158, 159]. Nevertheless, whether lipocalin-2 plays a role in the pathogenesis of obesity-related diseases has not been investigated so far.

Recent studies have reported the association between serum LCN2 concentrations and various metabolic parameters and inflammatory markers [158, 160, 161]. The study of Jang et al. provides the first clinical evidence demonstrating that serum concentrations of LCN2 are closely associated with obesity and its related chronic inflammation and metabolic complications. Patients with MetS showed higher levels of LCN2 than those without MetS. However, correlation between serum LCN2 concentration and the number of MetS components was not significant. Nonetheless, they suggest serum LCN2 as a useful biomarker for evaluating the outcomes in various clinical settings of obesity-related metabolic and cardiovascular disease [162].

LCN2 is expressed in different types of cells and it has been identified in chondrocytes [163]. In these cells, LCN2 expression was modulated by IL-1*β*, leptin, adiponectin, LPS, and dexamethasone [164]. In addition, the synovial fluid from patients with knee OA was found to be enriched with MMP-9/LCN2 complexes that have been involved in matrix degradation [165]. Recently, the group of Katano confirmed that synovial fluid levels of LCN2 were significantly higher in patients with RA than in those with OA. Through a proteome analysis, they showed that granulocyte macrophage colony-stimulating factor (GM-CSF) can contribute to the pathogenesis of RA by upregulating LCN2 in neutrophils, followed by the induction of a series of enzymes, such as cathepsin D, transitional endoplasmic reticulum ATPase (TERA), and transglutaminase 2 (tg2) in synoviocytes, which could contribute to the proliferation of synovial cells and infiltration of inflammatory cells inside the synovium [166]. Accordingly, lipocalin may regulate immune cell recruitment to the site of inflammation, a process essential for the controlled initiation, perpetuation, and resolution of inflammatory processes [167]. Finally, very recently, the NEIRID group had showed that nitric oxide boosts TLR-4 mediated lipocalin expression in chondrocytes, suggesting the existence of a feedback loop regulating the expression of this adipokine [168].

### 8.2. Chemerin

Chemerin, also known as TIG2 (tazarotene-induced gene 2) or RARRES2 (retinoic acid receptor responder 2), is an adipokine with chemoattractant activity. It is secreted as an 18 kDa inactive proprotein and it is activated by posttranslational C-terminal cleavage. Chemerin acts via the G-protein-coupled receptor chemokine-like receptor 1 (CMKLR1 or ChemR23) [169]. Chemerin and its receptor are mainly, but not exclusively, expressed in adipose tissue [170] and, for instance, dendritic cells and macrophages express chemerin receptor [171]. It has been implicated in immune [169] and metabolic homeostasis [170]. Chemerin expression correlates with BMI in humans and is upregulated in the adipose tissue of obese and T2DM sand rats (*Psammomys obesus*) [170]. IL-1*β* has been reported to induce chemerin expression in mouse adipocytes [172].

Bozaoglu et al. identified, for the first time, chemerin as a novel adipokine, which may play a role in the pathophysiology of obesity and MetS. They showed that plasma chemerin concentrations were strongly associated with BMI, plasma triglycerides, and blood pressure. These findings suggest that chemerin may play an important role in obesity and MetS. Moreover, it raises the possibility that chemerin may be of value as a biomarker for this disorder [108]. Recently, two studies determine chemerin levels in plasma and subcutaneous adipose tissue (SAT) in nascent MetS patients, without concomitant diabetes or CVD. In their studies, they made the observation that both plasma and SAT levels of chemerin were higher in subjects with nascent MetS, suggesting an early role of chemerin in the pathogenesis of MetS [95, 173].

Interestingly, chondrocytes express chemerin and its receptor [164, 174], and IL-1*β* is able to increase chemerin expression [164]. In the same way, Berg et al. have demonstrated that recombinant chemerin enhances the production of several proinflammatory cytokines (TNF-*βα*, IL-1*β*, IL-6, and IL-8), as well as different MMPs (MMP-1, MMP-2, MMP-3, MMP-8, and MMP-13), in human articular chondrocytes [174]. These factors have a role in the joint inflammation and degradation of the extracellular matrix by causing breakdown of the collagen and aggrecan framework and result in irreversible destruction of the cartilage in OA and RA. Furthermore, chemerin was recently detected in synovial fluid from OA and RA patients [175, 176]. The serum concentration of this adipokine correlated with disease severity in OA [176] and with disease activity in RA [177].

### 8.3. Omentin

Omentin is a 40 kDa protein secreted by omental adipose tissue that has previously been identified as intelectin, a new type of Ca^2+^-dependent lectin. It is highly and selectively expressed in visceral adipose tissue, and that might regulate insulin action by increasing insulin-mediated glucose uptake in human subcutaneous and omental adipocytes [178]. Plasma omentin levels and gene expression in adipose tissue decrease with obesity and correlate positively with plasma adiponectin and high-density lipoprotein levels and negatively with waist circumference, BMI, and insulin levels, all of which are markers of MetS [179]. Expression of the omentin gene was demonstrated in omental adipose tissue of patients with Crohn's disease, suggesting a role in chronic inflammatory diseases [180].

In two different studies, the group of Bremer determined omentin levels in nascent MetS patients. They observed that both plasma and subcutaneous adipose tissue levels of omentin were lower in subjects with nascent MetS, suggesting that lower secretion of omentin from SAT in subjects with nascent MetS establishes the presence of omentin deficiency in the syndrome as well [95, 173].

More recently, Šenolt et al. have demonstrated reduced levels of omentin in the synovial fluid of patients with RA compared with those with OA [181]. This finding suggests that this adipokine is likely to be involved in OA pathophysiology.

## 9. Conclusions

The relationships among metabolic syndrome, adipokines, and rheumatic diseases are complex, encompassing a variety of influences that include also cardiovascular function, metabolic status, biomechanics, and also behavioural aspects. A critical aspect that we have always borne in mind is that MetS is closely related to inflammation or chronic “low-grade inflammatory state” that will influence heavily the courses of rheumatic diseases. Thus, the first line approach to tackle MetS is the prevention of excessive weight gain across lifespan. Of course, this kind of approach should not be an individual task but rather should be a social priority. However, this strategy, in order to be effective, might require deep sociocultural changes, as well as international coordinated social instructions that right now are really far from being achieved.

## Figures and Tables

**Table 1:** Major adipokines and the effect of anti-TNF-*α* therapy.

Adipokines	RA	Anti-TNF-*α* therapy	Reference
Leptin	↑ or (—)	No effect in serum levels	[64]
Adiponectin	↑	No effect	[67]
Visfatin	↑	Reduction in serum levels	[68, 69]
		Not variation	[70]
Resistin	↑	Reduction in serum levels	[69, 71]
